# Construction of novel hypoxia-related gene model for prognosis and tumor microenvironment in endometrial carcinoma

**DOI:** 10.3389/fendo.2022.1075431

**Published:** 2022-12-15

**Authors:** Junfeng Chen, Guocheng Wang, Donghai Zhang, Xiaomei Luo, Da Zhang, Yongli Zhang

**Affiliations:** ^1^ Shanghai Key Laboratory of Maternal Fetal Medicine, Shanghai Institute of Maternal-Fetal Medicine and Gynecologic Oncology, Shanghai First Maternity and Infant Hospital, School of Medicine, Tongji University, Shanghai, China; ^2^ Department of Gynecological Oncology, Changchun Center Hospital, Changchun, Jilin, China

**Keywords:** endometrial cancer, hypoxia, prognostic, tumor microenvironment, risk score

## Abstract

**Introduction:**

Endometrial cancer is currently one of the three most common female reproductive cancers, which seriously threatens women’s lives and health. Hypoxia disrupts the tumor microenvironment, thereby affecting tumor progression and drug resistance.

**Methods:**

We established hypoxia-related gene model to predict patient prognosis and 1-, 3-, and 5-year overall survival rates. Then, the expression level of hypoxia-related genes and survival data were extracted for comprehensive analysis by Cox regression analysis, and the model was established.

**Results:**

We analyzed the survival and prognosis of patients in the high and low-risk groups. The Kaplan-Meier curve showed that the low-risk group is associated with a better survival rate. The 1-, 3-, and 5-year AUC values of the model were 0.680, 0.698, and 0.687, respectively. Finally, we found that LAG3 may be a potential immune checkpoint for endometrial cancer.

**Conclusion:**

We found four hypoxia-related genes (ANXA2, AKAP12, NR3C1, and GPI) associated with prognosis. The hypoxia-related gene model can also predict prognosis and tumor microenvironment in endometrial cancer.

## Introduction

Endometrial cancer is the most prevalent malignant tumor in gynecology (1). In China, endometrial cancer has now become the second-most frequent gynecological cancer (2, 3). There were about 69,000 new cases of endometrial cancer diagnosed and 16,000 deaths in 2015, with an annual growth rate of 3.7 percent (2, 3). It is the most common cancer of the female reproductive organs and the second most malignant tumor of the female reproductive system in China, after cervical cancer (1). Despite tremendous improvements in surgical techniques and medical treatment, the survival rate of Uterine Corpus Endometrial Carcinoma (UCEC) patients has not improved efficiently. However, a growing number of studies have found that the high mortality and poor prognosis of UCEC are linked to the tumor microenvironment (TME) (4).

Drug resistance is the primary cause of treatment failure in end-stage cancer (5). The tumor microenvironment is one of the primary causes of drug resistance (6). Recent research has also shown that TME is dominated by hypoxia in cancers (6–8). Uncontrolled multiplication of tumors reduces oxygen availability and leads to inadequate blood supply (9). Hypoxia is a common microenvironmental trait in nearly all solid tumors (10). Abnormal angiogenesis, desmoplasia, and inflammation are all promoted by an aberrant vasculature and a hypoxic microenvironment, all of which contribute to tumor growth and therapeutic resistance (10).

The extremely hypoxic environment causes significant changes in the tissues and cells in the TME (11). Hypoxia affects basic mechanisms of pre-mRNA splicing, including miRNA synthesis and maturation, splicing factor expression and activity, spliceosome assembly and intracellular localization, as well as mRNA structure and elongation rate (12). Hypoxia, as a whole, stimulates the growth of immunosuppressive cells (MDSC, Treg cells, macrophages, and immunosuppressive cytokines) in the TME (13). However, it also impairs the adjustment of anti-tumor immunity by reducing the killing, survival, and migration of effector cells (NK cells, CD4^+^, and CD8^+^ T cells) (13).

Many clinical trials on immunotherapy for different malignancies have been conducted in recent years, but they are limited to the efficacy of therapeutic treatment established on UCEC (14, 15). Bioinformatics-based exploration has recently emerged as a viable strategy for clinical development in modern oncology (16, 17). It has practical application value by detecting the altered expression of model genes in endometrial cancer tissues and converting model genes into risk scores to predict patient prognosis (18, 19). In this study, we built a hypoxia-related gene model to predict endometrial cancer overall survival in the TCGA.

## Materials and methods

### Raw data sources and data preprocessing

We analyzed the transcriptome RNA-seq data (normal count: 23, tumor count: 552) and clinical data (cases: 548) downloaded from The Cancer Genome Atlas (TCGA, http://cancergenome.nih.gov/). Based on the limma R package, we utilized the Wilcoxon test to sort out the meaningful differentially expressed genes (DEGs). The |log2-fold change (FC)| > 1 and the adjusted *p* < 0.05 were chosen as the cut-off criteria. The tumor immune gene set and hypoxic marker gene set were downloaded from the Tracking Tumor Immunophenotype website (http://biocc.hrbmu.edu.cn/TIP/index.jsp) and the GSEA website (https://www.gsea-msigdb.org/gsea/index.jsp), respectively.

### Construction of the protein–protein interaction networks

The hypoxia gene set was utilized to extract hypoxia-related genes (HRGs) expression levels from transcriptome RNA-seq data of TCGA. The PPI networks were created by the STRING database (http://string-db.org) and were visualized and integrated. Based on the number of interrelationships, the Cytoscape software (https://cytoscape.org/) platform was used to examine the correlation of HRGs in the protein interaction relationship network, and then the top 50 genes with the maximum number of adjacent nodes were analyzed as the key core genes.

### The constitution of the risk model

We first used univariate Cox regression on key core genes to discern HRGs with prognostic outcome. The Lasso regression was then used to guarantee that the multidimensional model would not overfit. The multivariate Cox regression was to determine the genes used to construct the model and confirm their coefficients. Each patient’s risk score was calculated from the retrieved genes, and patients were divided into low and high hypoxic risk groups based on the median risk score. The risk score formula was defined as:

Risk Score = Expression gene1 × Coefficient gene1 + Expression gene2 × Coefficient gene2+… +Expression geneN × Coefficient geneN,

where N = 4 indicates the expression levels of a total of the four HRGs. Gene Expression represented the expression levels of each HRG. The coefficient gene represented the corresponding multivariate Cox regression coefficients.

### The prognostic efficacy of the risk model

Subsequently, we examined whether the risk value was correlated with patient overall survival (OS). The Kaplan-Meier method was used to calculate OS for patients in the low- and high-risk groups. We used univariate, Lasso, and multivariate COX regression analyses to determine whether risk scores could be distinguished from other routine clinical features as an independent prognostic factor for patients. The *p*-value < 0.05 was considered statistically significant, and the 95 percent confidence interval was also used to determine the hazard ratio (HR). ROC curves constructed using the survival ROC R package evaluate the accuracy and reliability of the risk model for predicting patient OS. To better assess the survival probability of 1, 3, and 5 years for UCEC patients, the nomogram model with four genes was constructed based on the results of both univariate and multivariate analyses. We also plotted survival curves to confirm if all genes differentiated between high and low grouping prognosis considerably.

### Gene ontology, kyoto encyclopedia of genes and gene set enrichment analysis

Using the clusterProfiler R package, bar charts and bubble charts predicted the probable functions of HRGs *via* GO and KEGG. We showed the main GO and KEGG pathways depending on *p*-value < 0.05 and showed the results in bar charts and bubble charts using the ggplot2 R package. To investigate the differences in biological function in the HRGs between low- and high-risk groups, GSEA was used to enrich the Molecular Signatures Database (MSigDB) (h.all.v7.4.symbols.gmt [Hallmarks]). For each analysis, 1000 permutations of gene sets were performed. Statistical significance was defined as NOM *p*-value < 0.05 and NES > 1.

### Evaluation of immune cell type components

CIBERSORT (https://cibersort.stanford.edu/) is a tool that determines the proportion of various cell subtypes in mixed cell samples. It’s a standard way of estimating and studying immune cell infiltration. We used it to analyze the proportions of 22 immune cell subtypes, including B cells, T cells, and NK cells, in the high- and low-risk groups according to the previously calculated median value of each immune-related cell infiltration. The sample’s total relative composition of all immune cell types was equal to one.

### Immune function and immunosuppressive genes

ssGSEA is used to quantify the tumor-infiltrating cell-related pathways (MHC class I, CCR, APC co-stimulation, APC co-inhibition, HLA, check-point, cytolytic activity, inflammation promoting, parainflammation, T cell co-inhibition, type I_IFN response, T cell co-stimulation, and type II_IFN response). It could be seen if there was a link between the two risk groups and the immunological state. We investigated genes in the model related to immune checkpoints, m6A-related genes and several immunosuppressive genes in low- and high-risk groups. Heat maps and histograms showed the difference in expression using the ggplot2 packages. The TIP was used to acquire related-gene signatures.

### Validation of the prognostic gene signature

UCEC (pathologically confirmed by two independent senior pathologists) and tumor-adjacent normal tissues were obtained from the Department of Biobank of Shanghai First Maternity and Infant Hospital. All patients were informed, and formal informed permission was obtained. This study was approved by our hospital’s Protection of Human Subjects Committee and was performed according to the relevant guidelines. Specimens were acquired with written informed consent from patients at the Shanghai First Maternity and Infant Hospital affiliated with Tongji University. The study was conducted in accordance with the Declaration of Helsinki. The TRIzol (Invitrogen, Carlsbad, CA, USA) reagent was used to extract RNA from tissue samples. The QuantiTect Reverse Transcription Kit (QIAGEN, Valencia, CA, USA) was used to reverse-transcribe RNA into cDNA. SYBR-Green (Takara, Otsu, Shiga, Japan) was used to quantify real-time PCR results, and levels were standardized to ACTB levels. The primers for five genes are listed in **Table 1**. Western blot analysis was performed using antibodies against rabbit monoclonal antibody-anti-human GAPDH (1:5000, AC001), ANXA2(1:1000, A11235), GPI (1:1000, A6916), NR3C1 (1:1000, A19583) from ABclonal, and rabbit monoclonal antibody-anti-human AKAP12 (1:500, 25199-1-AP) from Proteintech, followed by incubation with horseradish peroxidase (HRP)-coupled rabbit secondary antibody (1:1000, #7074, Cell Signaling Technology). We verified the expression of four genes in normal and tumor tissue by utilizing the Human Protein Atlas (HPA) database.

**Table 1:** Primer sequences.

	Primer sequences(5′-3′)
ANXA2 forward	CCGGCTCTGCTCAGCATTTG
ANXA2 reverse	GCTATGCTACAAGATAACCTGGGC
AKAP12 forward	CTGTCTGCCGTCAATGGTGTA
AKAP12 reverse	TGAAGCAGGGATCTGTTCGAT
NR3C1 forward	ACAGCATCCCTTTCTCAACAG
NR3C1 reverse	AGATCCTTGGCACCTATTCCAAT
GPI forward	CAAGGACCGCTTCAACCACTT
GPI reverse	CCAGGATGGGTGTGTTTGACC
ACTB forward	CATGTACGTTGCTATCCAGGC
ACTB reverse	CTCCTTAATGTCACGCACGAT

## Results

### Hypoxia-related genes in UCEC

**Figure 1:**
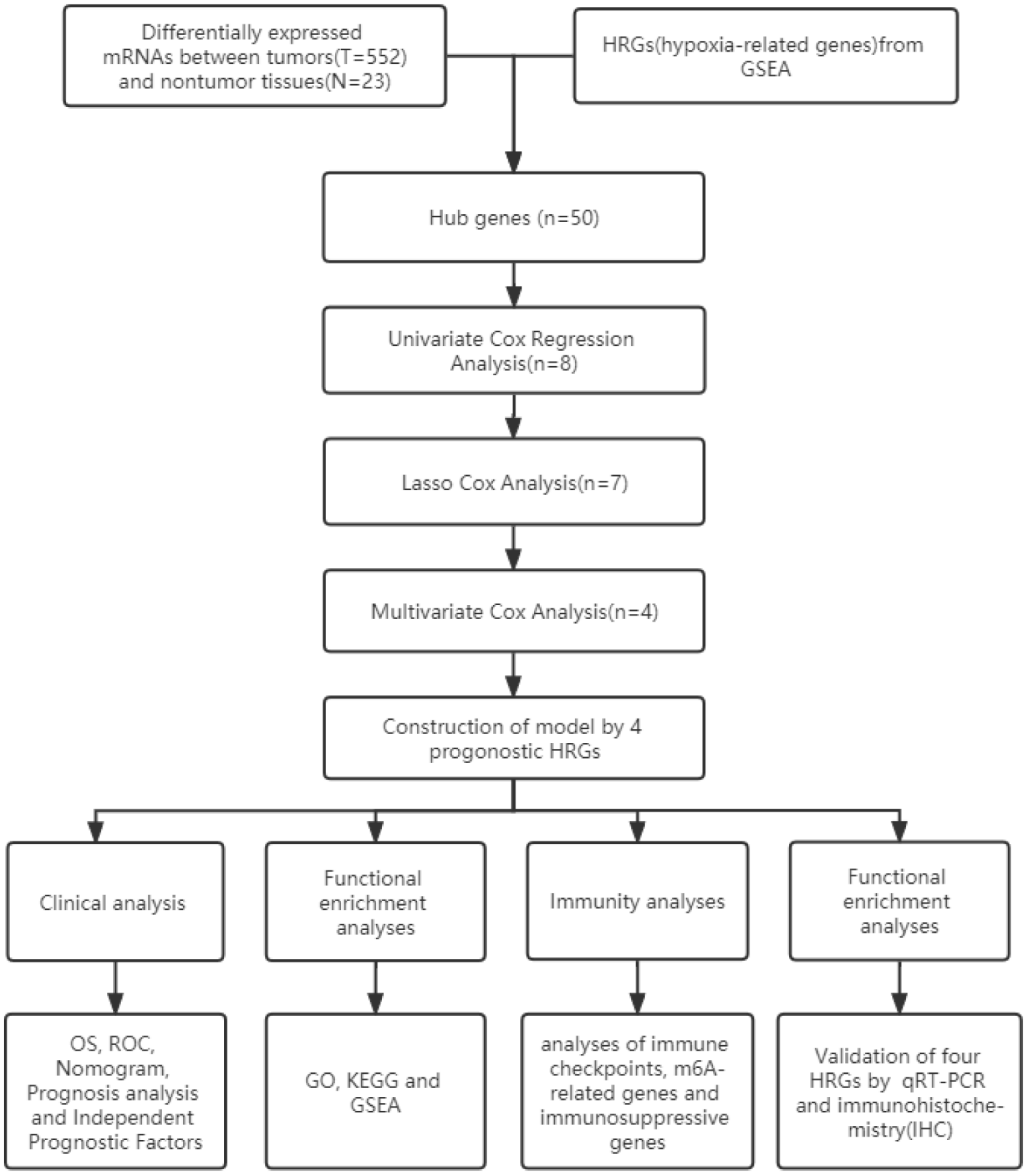
Flow of this study.

**Figure 2:**
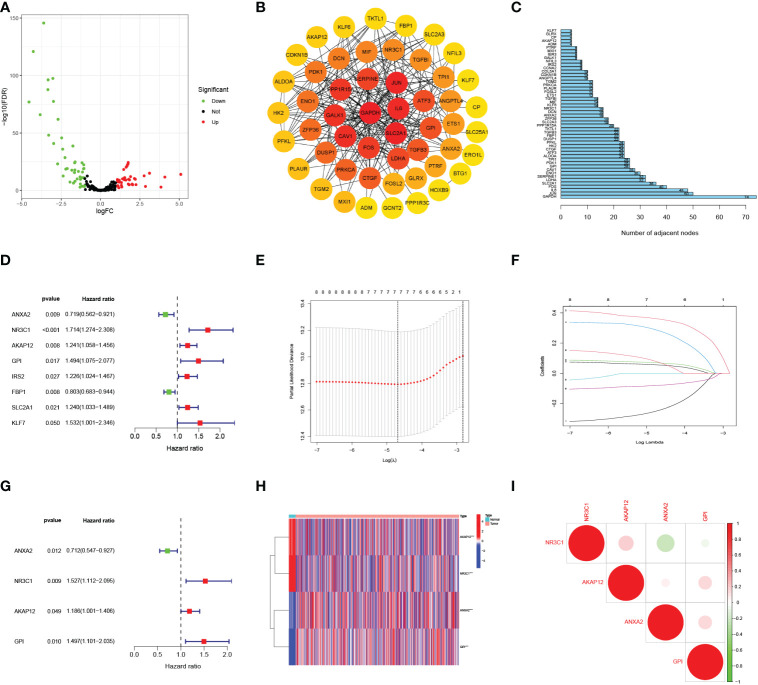
Selecting hypoxia-associated key core genes and constructing the model by Univariate, Lasso, and Multivariate Cox Analysis. **(A)** Volcano map of differential hypoxia-associated genes, absolute log2-fold change (FC) > 1, and adjusted p value < 0.05 were used as screening criteria for differential genes. **(B)** Protein-protein interaction network containing these genes. **(C)** The top 50 genes were selected based on the number of nodes. **(D)** Univariate Cox regression analysis identified candidate genes with the *p*-value < 0.05. **(E, F)** Establishing a hypoxia prognostic model by LASSO regression analysis. **(G)** Multivariate Cox regression analysis of hypoxia-related genes. **(H)** Heatmap of differential hypoxia-associated genes in normal samples and endometrial cancer. **(I)** Correlations between the genes included in the risk model. *** represents a p value < 0.001.

**Figure 1** shows the work flow diagram for this study. The clinical information of patients with endometrial cancer was shown in **Table 2**. The volcano plot showed a differential distribution of gene expression levels between the normal and tumor groups (**Figure 2A**). We then used the PPI network analysis software, the STRING online database, and CYTOSCAPE to establish the relationship between these genes (**Figure 2B**). The top 50 genes with the highest levels of interaction were selected as key genes. The results showed that the key genes, including *GAPDH, JUN, IL6, FOS, SLC2A1, LDHA, CAV1, GPI*, et al. (**Figure 2C**).

**Table 2:** Clinical information of Uterine Corpus Endometrial Carcinoma cohort.

Clinical features	TCGA-UECE(N=548)	
	No	%
OS		
0	461	84.12
1	87	15.88
Age		
<=60	209	38.35
>60	336	61.65
Stage		
I	339	62.20
II	52	9.54
III	124	22.75
IV	30	5.50
Grade		
I	99	18.07
II	122	22.26
III	316	57.66
IV	11	2.01

### The risk score model was built based on core HRGs

By the univariate Cox analysis, eight HRGs (*ANXA2, NR3C1, AKAP12, GPI, IRS2, FBP1, SLC2A1, KLF7*) were shown to be strongly related to patient OS prognosis (**Figure 2D**). According to the optimum λ value (**Figures 2E, F**), four core HRGs of them were chosen to build the model by the multivariate Cox regression analysis (**Figure 2G**, **Supplementary Table 1**). The heatmap displayed contrasts in the expression of four core HRGs in the normal and tumor groups (**Figure 2H**). We additionally investigated the correlation between four genes identified as predicting patient prognosis in the risk model (**Figure 2I**). Red and green represented positive and negative relationships, respectively. Simultaneously, Spearman correlation analysis revealed no significant correlation between the four HRGs. The risk score was calculated as: Risk Score = (-0.340*ANXA2 expression level) + (0.423*NR3C1 expression level) + (0.171*AKAP12 expression level) + (0.402*GPI expression level).

### Patients with various risk scores had varying prognoses

**Figure 3:**
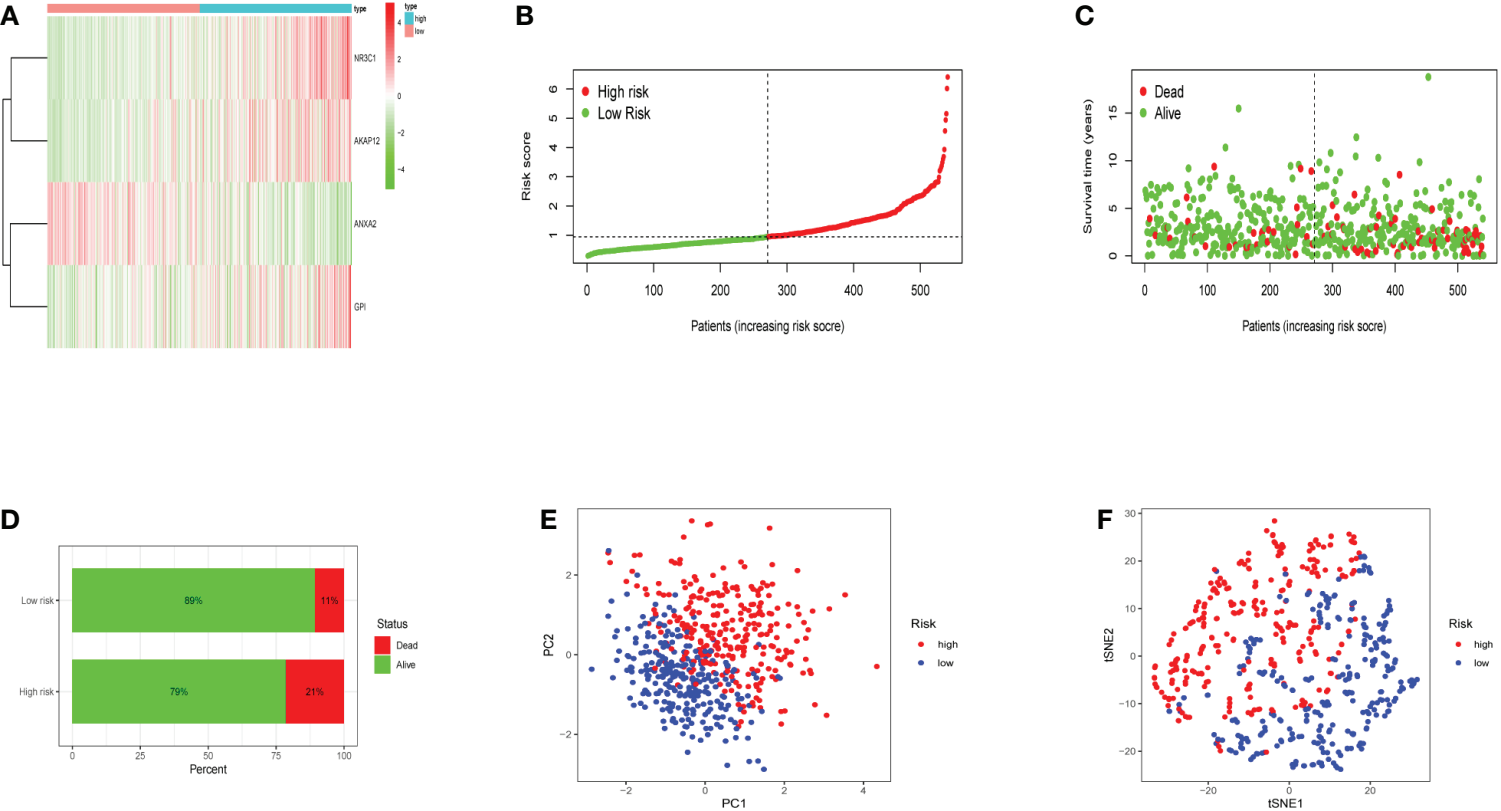
The expression levels of the four genes included in the model and the prediction of patient risk in the hypoxia model. **(A)** Heatmaps of four gene expression levels in the risk model for the high- and low-risk groups. **(B)** Patient risk scores in the high- and low-risk groups. **(C)** Survival rates in the high- and low-risk groups. **(D)** Patient survival in the high- and low-risk groups. **(E, F)** Principal component analysis (PCA) plot and t-distributed stochastic neighbor embedding (t-SNE) for the different gene expression patterns of samples.

The heatmap revealed that the expression of three genes (*NR3C1, AKAP12*, and *GPI*) increased substantially with risk scores (**Figure 3A**). Risk scores were observed in low- and high-risk groups, and hypoxia risk scores increased with increased risk levels of patients (**Figure 3B**). **Figures 3C, D** revealed that patients in the high-risk group had a significantly higher death rate than those in the low-risk group, with significant prognostic differences. All the above results explain that as hypoxia risk scores rise, mortality also increases in endometrial cancer patients. Patients with various risks were also well segregated into two groups by principal component analysis (PCA) and t-distributed stochastic neighbor embedding (t-SNE) analysis (**Figures 3E, F**).

**Figure 4:**
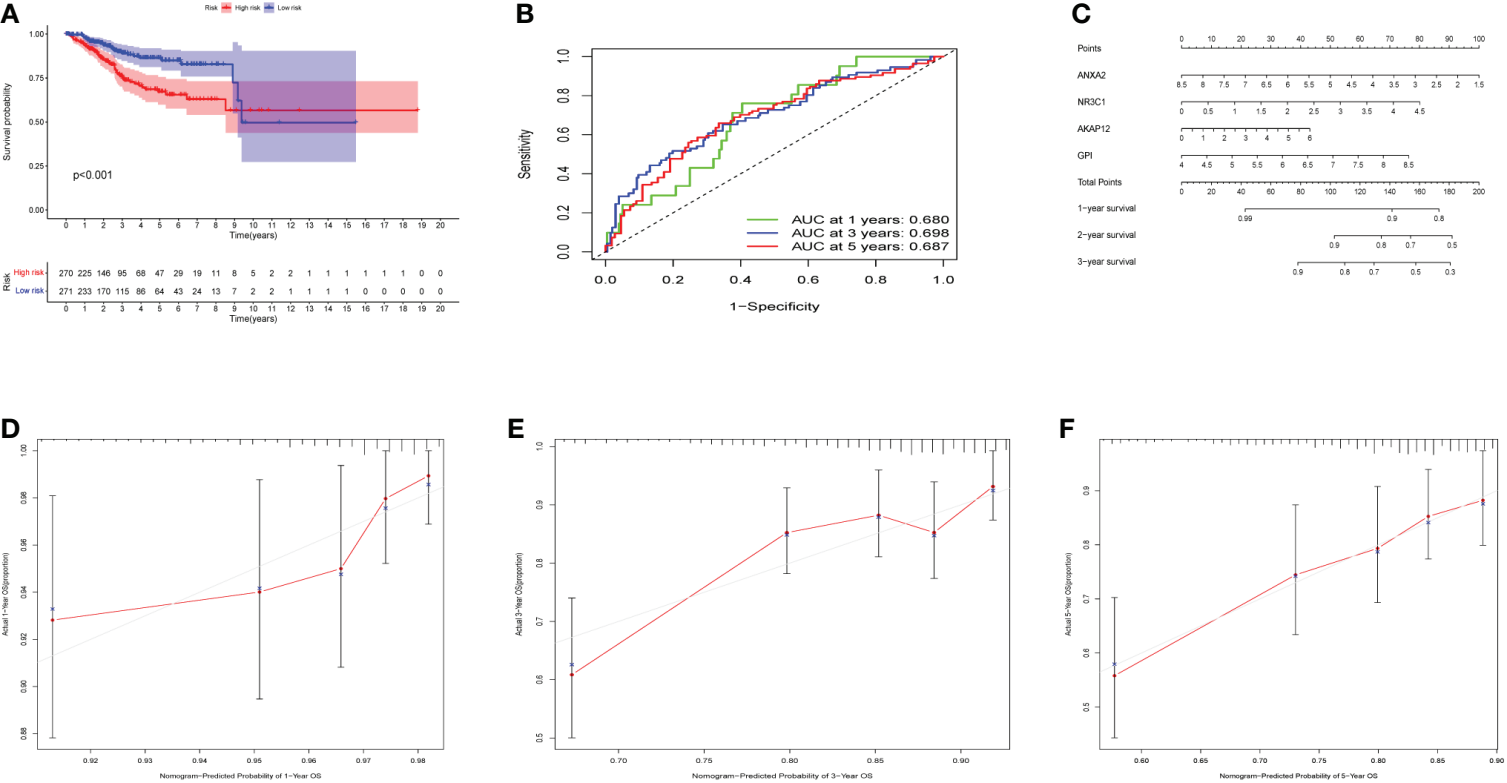
Effects of the hypoxia model on patient prognosis. **(A)** Kaplan–Meier survival curves for patients, stratified according to risk scores; comparison of the median survival time with log-rank tests (*p* < 0.001). **(B)** Receiver operating characteristic curve analysis of the prognostic accuracy of the model. **(C)** Nomogram based on four genes for predicting the prognostic survival rate of patients. **(D-F)** Calibration curves of the nomogram for predicting the survival outcomes at 1-, 3-, and 5-years.

The OS curve showed that high-risk hypoxia scores were related to worse prognostic outcomes as compared to low-risk hypoxia scores (**Figure 4A**). ROC curves showed AUCs of 0.680, 0.690, and 0.687 that were predicted by the 1, 3, and 5-year OS, respectively (**Figure 4B**). ROC demonstrated that AUC at 1, 3, and 5-year was higher than 0.6, indicating our risk model’s predictive potential (**Figure 4B**). In addition, we established the nomogram to further calculate the survival probability for each patient (**Figure 4C**). Each patient’s 1, 3, and 5-year survival rates might be estimated based on the expression of four genes. **Figures 4D-F** show separately how the 1, 3, and 5-year curve levels well overlap the calibration curve. The outcome indicated that it could evaluate the prediction accuracy of the model and that the risk model could better predict the prognosis of endometrial cancer.

### Hypoxia risk factors were prognostic independently from clinical characteristics

**Figure 5:**
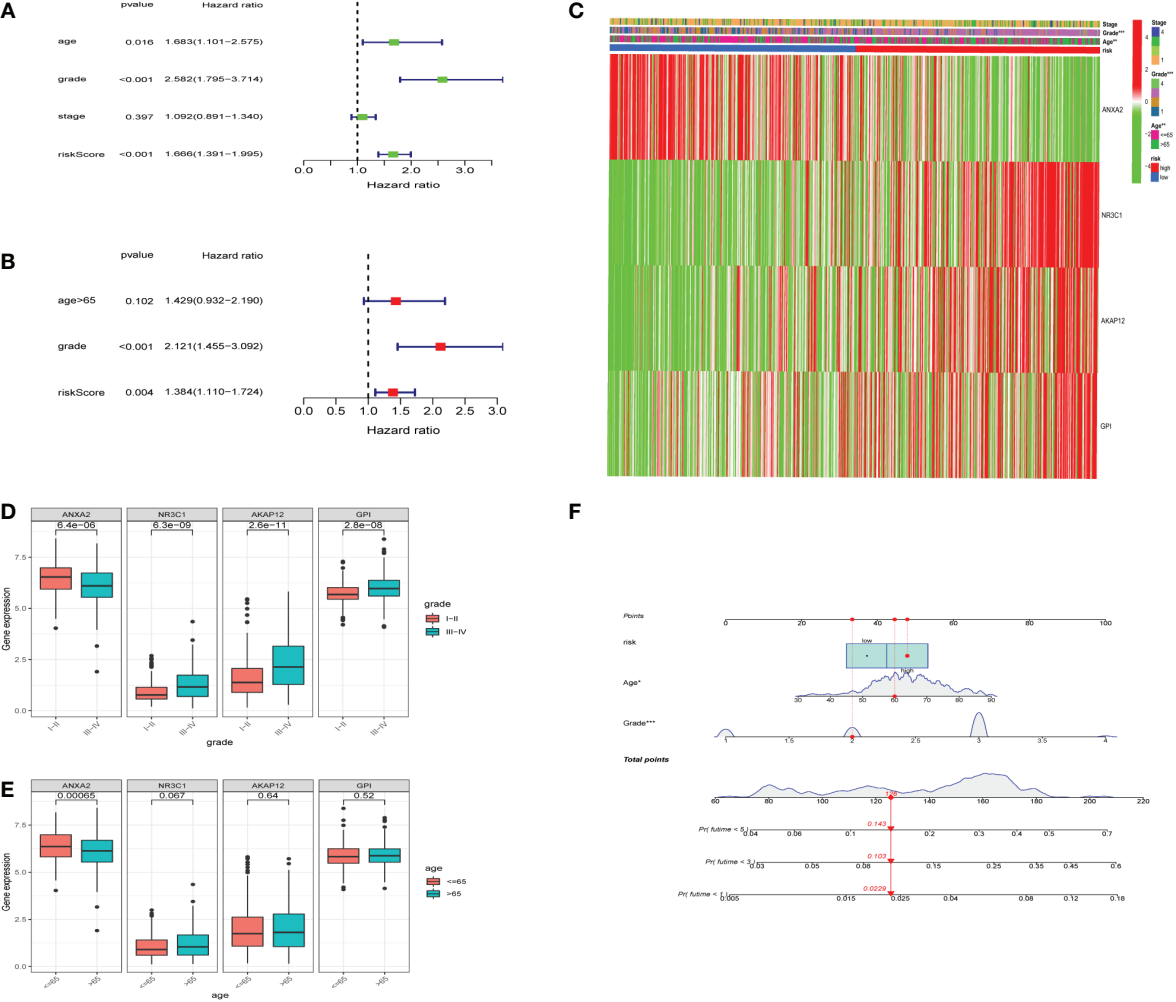
Relationship between the risk model and clinical factors. **(A)** A single-factor prognostic analysis included age, grade, stage, and the risk scores of patients with endometrial cancer. **(B)** Multifactor prognostic analysis included age, grade, and the risk scores of patients with endometrial cancer. **(C)** Heatmap (green: low expression; red: high expression) for the connections between clinical characteristics (stages, grades, and ages) of patients in high-risk and low-risk samples. **(D, E)** Comparisons of the expression levels of various genes in the hypoxia model for different ages and grades. **(F)** Nomogram based on risk, age, and grade for predicting the prognostic survival rate of patients. * represents a p-value < 0.05, ** represents a p-value < 0.01, *** represents a p-value < 0.001.

To investigate whether the hypoxia risk score could be used as an independent prognostic factor in predicting the survival of UCEC patients, we employed both univariate and multivariate Cox regression analyses on the four HRGs to compare clinicopathological features (age, gender, grade, and stage) (**Supplementary Table 2**). The age, grade, stage, and risk score were all connected to overall survival according to the univariate Cox regression (**Figure 5A**). Then we found that the hypoxia risk score was independently related to the difference in overall survival of UCEC patients (**Figure 5B**). Two analyses were as follows: our panel might be regarded as an independent prognostic indicator for UCEC. The heatmap depicted the expression characteristics of the four genes in various risk groups and their correlation to clinical factors (stage, age, grade) (**Figure 5C**). We observed that the risk score was merely associated with age and tumor grade. We studied how the four genes are expressed differently in patients of diverse ages and grades (**Figures 5D, E**). All four genes were expressed at different levels in patients of various grades (**Figure 5D**). Based on the risk score, age, and grade, the 1, 3, and 5-year survival rates of each patient could be predicted by the nomogram (**Figure 5F**).

### GO, KEGG and GSEA

GO indicated that the most enriched GO terms were BP (biological process) including the monosaccharide metabolic process, CC (cellular component) including collagen-containing extracellular matrix, and MF (molecular function) including monosaccharide binding (**Supplementary Figures 1A, B**). KEGG pathways were mainly related to HIF-1 signaling pathway, Glycolysis/Gluconeogenesis and Carbon metabolism (**Supplementary Figures 1C, D**). According to GSEA analysis, the potential signaling pathways such as E2F targets, G2M checkpoint, Mitotic spindle, Mtorc1 signaling, MYC targets, KRA signaling, and hypoxia were considerably enriched in high-risk groups (**Supplementary Figure 2**).

### Immune cell infiltration

**Figure 6:**
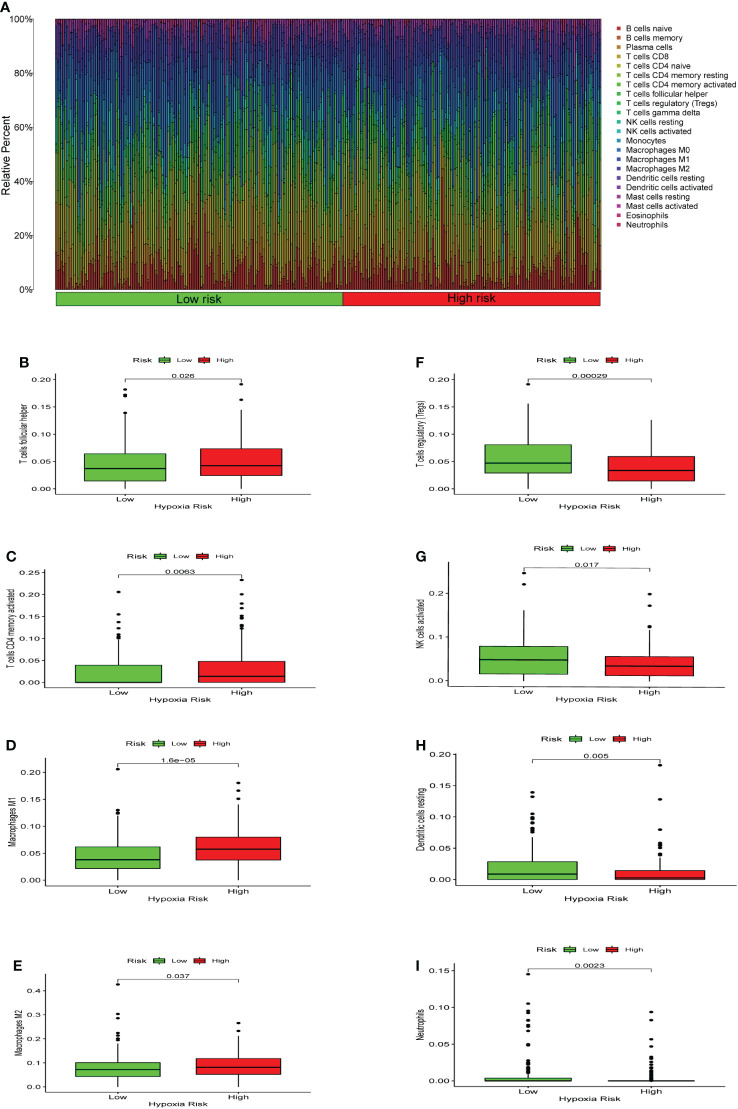
Enrichment of hypoxia pathways and infiltration of hypoxia-related immune cells. **(A)** A bar chart of hypoxia risk and immune cell infiltration. **(B-I)** Immune cells whose infiltration is significantly associated with the risk of hypoxia (p < 0.05).

We used CIBERSORT to evaluate the immune cell types and immune cell infiltration rates of the high-risk group and the low-risk group. (**Figure 6A**). There were significant differences in eight immune cells in the high-risk and low-risk groups. The results showed that the proportions of T cells CD4 memory activated (*p* = 0.0063), macrophages M1 (*p* = 1.6*10^-5^), macrophages M2 (*p* = 0.037), and T cells follicular helper (*p* = 0.026) were considerably higher in the high risk of hypoxia group (**Figures 6B-E**). However, in the high hypoxia risk group, the levels of dendritic cells resting (*p* = 0.005), neutrophils (*p* = 0.0023), NK cells activated (*p* = 0.017), and T cell regulatory (Tregs) (*p* = 0.00029) were markedly lower (**Figures 6F-I**). These data revealed that immune cell infiltration was substantially associated with hypoxia risk (*p* < 0.05), indicating that research into hypoxia is critical for future immunotherapy in tumor patients.

### Immune function and immunosuppressive genes

**Figure 7:**
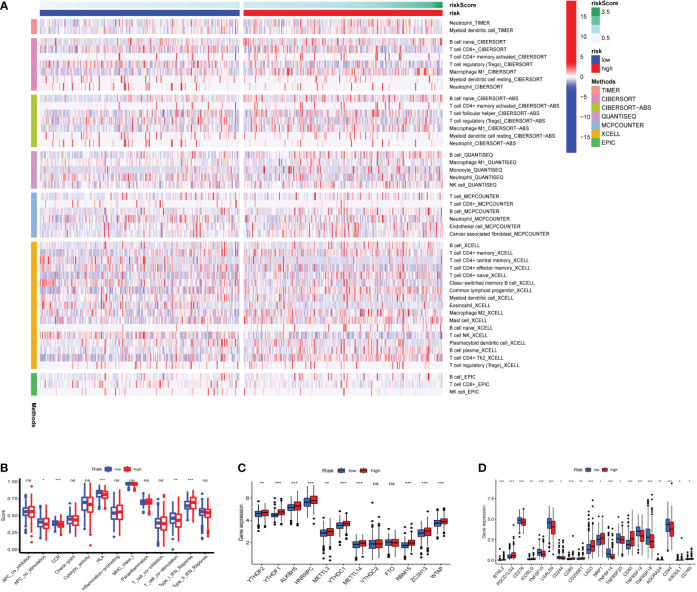
Heatmap for immune cells and their related functions, m6A-related genes, immune checkpoints **(A)** Heatmap for immune cells based on TIMER, CIBERSORT, CIBERSORT−ABS, QUANTISEQ, MCPCOUNTER, XCELL, and EPIC algorithms between two risk groups. **(B)** The immune cells related functions **(C)** m6A-related genes **(D)** immune checkpoints. ns represents a p value >0.05, * represents a p value < 0.05, ** represents a p value < 0.01, and *** represents a p value < 0.001.

**Figure 8:**
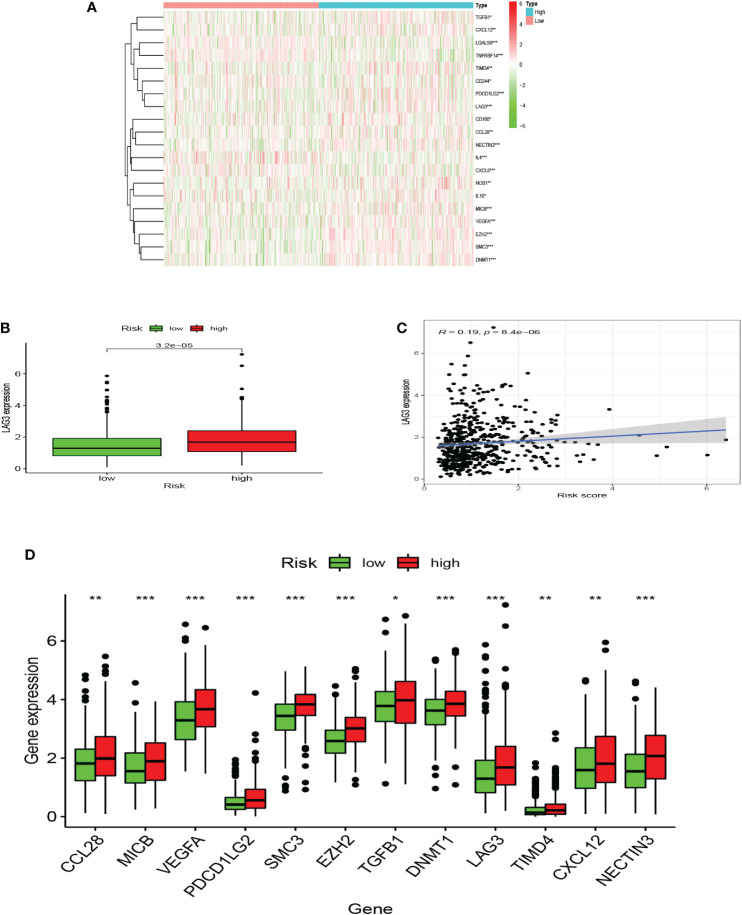
A microenvironment with a high risk of hypoxia tends to be immunosuppressive. **(A)** The heatmap displays the expression of the gene set involved in the negative regulation of anti-tumor immunotherapy in the low and high hypoxia risk groups. **(B)** The expression levels of LAG3 in high and low hypoxia risk groups. **(C)** Correlation between the expression of LAG3 and hypoxia risk score. **(D)** The expression of tumor immunosuppressive genes in the low and high hypoxia risk groups. (*P < 0.05, **P < 0.01 and ***P < 0.001).

Seven algorithms, including TIMER, CIBERSORT-ABS, QUANTISEQ, XCELL, MCPCOUNTER, EPIC, and CIBERSORT, were used to create a heatmap of tumor immune cell infiltration (**Figure 7A**). By single-sample gene set enrichment analysis (ssGSEA), the difference in immune cell activities revealed that CCR, HLA, and type I IFN response were all significantly different between the two risk groups (**Figure 7B**). We employed Gene Set Variation Analysis (GSVA) to investigate the differences in key immune checkpoint expression and m6A-related gene expression between the two risk groups since they are important in immunotherapy. Except for *FTO* and *YTHDC2*, most m6A-related gene expression differed markedly between the two risk groups (**Figure 7C**). Many immune checkpoint genes were shown to be substantially different between the two groups according to the boxplot (**Figure 7D**). In clinical treatment, however, these genes were rarely utilized as genes of immune checkpoint. We examined the commonly used five immune checkpoints (*PD-1, PD-L1, TIM3, CTLA-4*, and *LAG3*) in clinical treatment (**Supplementary Figure 3**). Only the *LAG3* expression levels differed substantially between the high- and low-risk groups. Following that, we depicted a box plot and a correlation curve to show the relationship between the expression levels of this gene and the risk score. The heatmap of the expression of gene sets involved in the negative regulation of anti-tumor immunotherapy was shown in **Figure 8A**. The box plot showed that low- and high-risk groups have different levels of gene expression (**Figure 8B**). The correlation curve revealed that the expression level of this gene was positively connected with the patient’s risk score (**Figure 8C**). **Figure 8D** reflected that most genes enriched in negatively regulated genes were higher in the high-risk score group, and the high hypoxia risk tended to favor the immunosuppressive microenvironment. It meant that patients with high hypoxia risk became insensitive to immunotherapy and had a poor immunotherapy outcome.

### Differential expression of markers was validated in an independent cohort

**Figure 9:**
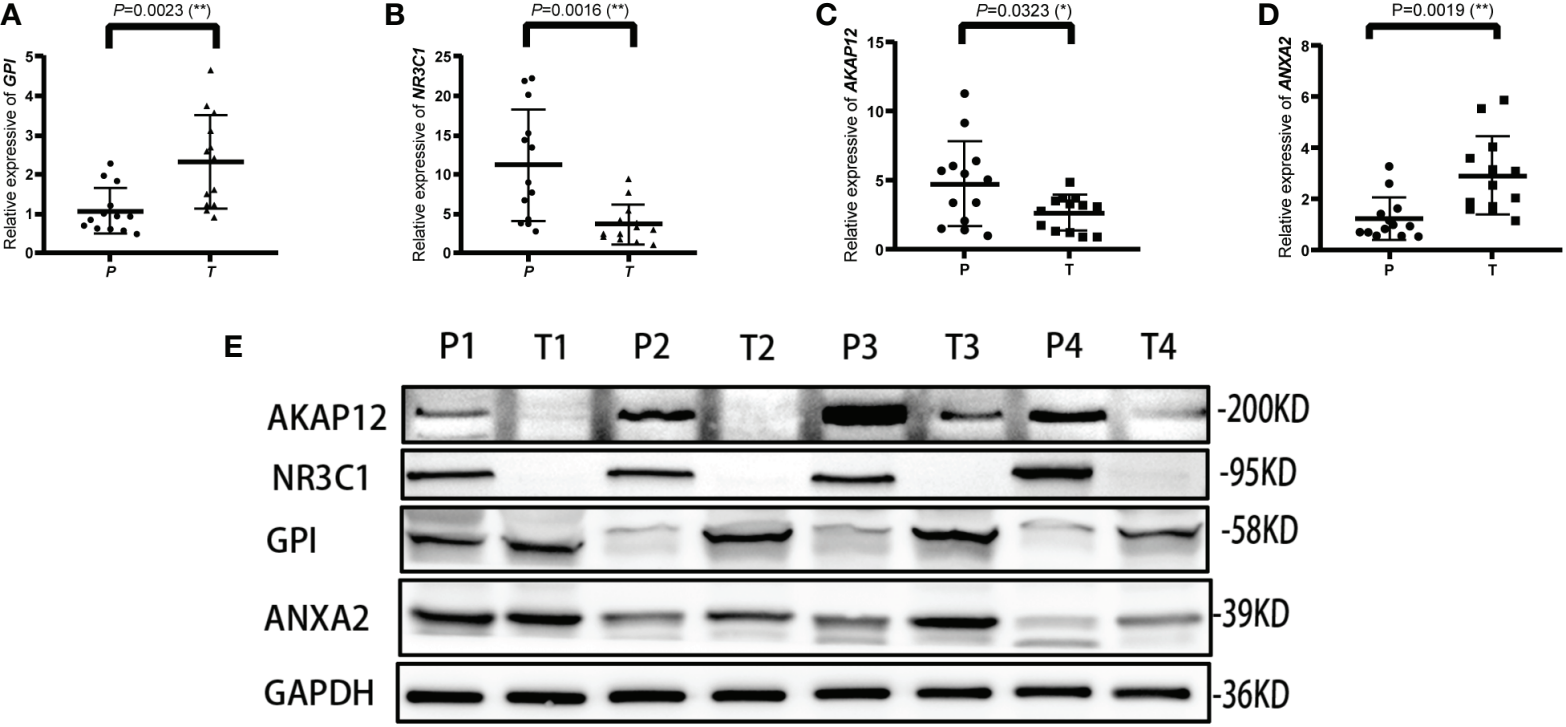
Validation of the relative expression level of *ANXA2, AKAP12, NR3C1* and *GPI* in para-carcinoma tissues (P) and tumor tissues (T) using qRT-PCR **(A–D)** and western blot **(E)**. * represents a p value < 0.05, ** represents a p value < 0.01.

**Figure 10:**
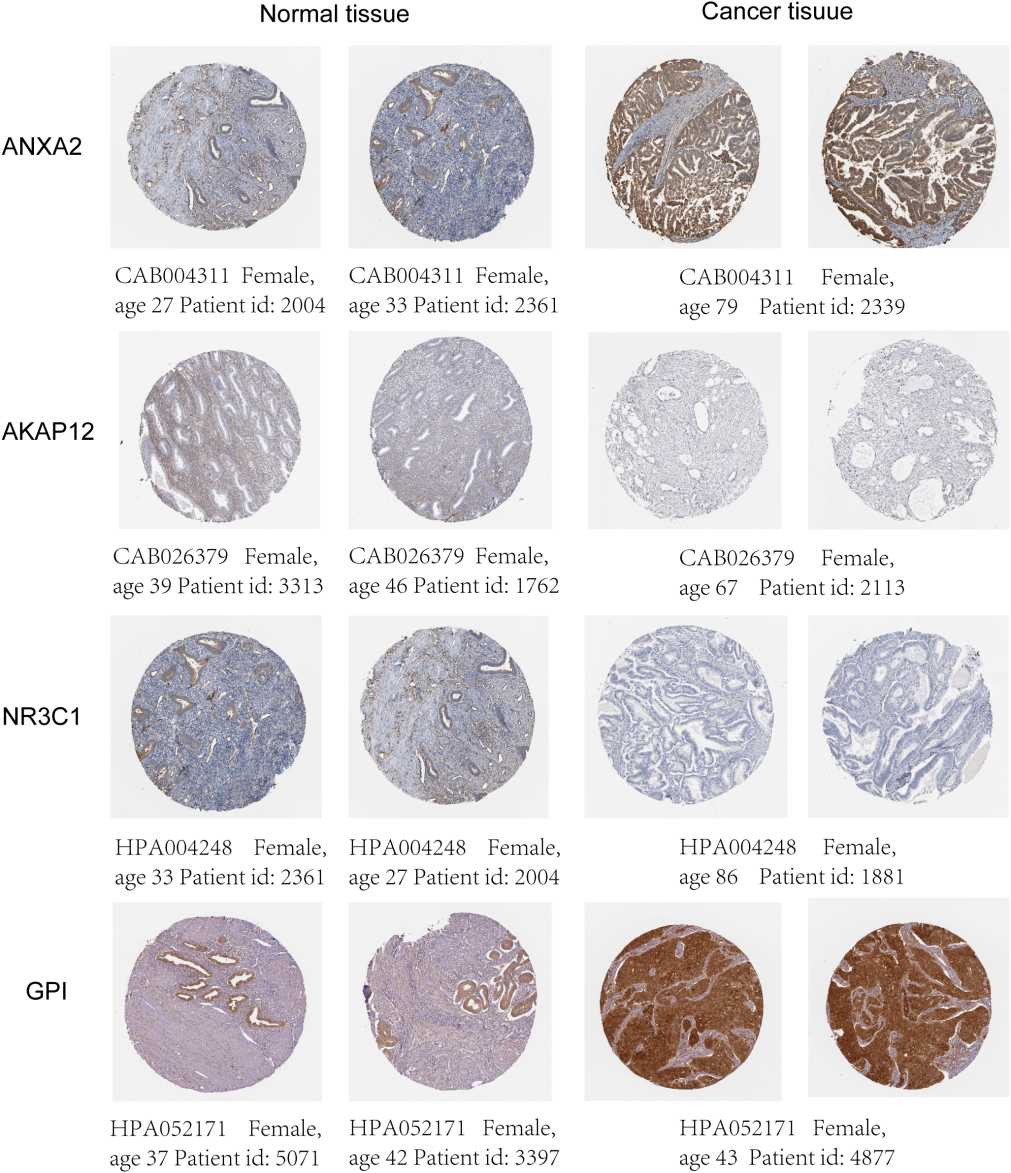
Differences in protein expression of the four HRGs in endometrial tissues from HPA.

The mRNA and protein levels of the four genes were subsequently determined using the RT-PCR experiment, which showed that *GPI* and *ANXA2* were highly expressed in UCEC compared to adjacent normal endometrial tissue, but *NR3C1* and *AKAP12* were downregulated (**Figures 9A-E**). In addition, we further explored the protein expression encoded by the four genes in endometrial cancer tissues. As shown in **Figure 10** from HPA database, *ANXA2* and *GPI* were strongly positive in UCEC tissues when compared with corresponding expression levels in non-tumor tissues. In contrast, *AKAP12* and *NR3C1* showed strong positivity in normal liver tissues.

## Discussion

Endometrial cancer is one of the most frequent gynecological cancers in the world and it affects roughly 420,000 women globally each year, with an approximated 76,000 women dying from it (20, 21). Although the impact of surgical therapy and medical treatment of endometrial cancer has improved in recent years, both the incidence and mortality of cancer are on the rise (22, 23). Hypoxia usually causes a lack of oxygen and nutrients in a variety of solid tumors, as well as problems with drug delivery. Tumor invasiveness, angiogenesis, and metastasis are all supported by hypoxia heterogeneity. These variables reduce anticancer medication therapeutic effectiveness and can be a barrier to progressing drug leads beyond the early phases of preclinical studies (24, 25). As a result, we constructed a model based on HRGs, investigated tumor-infiltrating immune cells in the prognosis of endometrial carcinoma, the tumor immune checkpoints, and m6A-related gene expression, and identified promising biomarkers and therapy targets for UCEC to assess prognostic and therapeutic efficacy.

In recent decades, development of Whole Genome Sequencing (WGS) has made biological data analysis easier, enabling the rapid growth of innovative treatments. Hypoxia is a significant characteristic of cancer. Previous research has found out how oxygen affects particular forms of cancer and how it might be used to predict prognosis, such as in oral squamous cell carcinoma (26), hepatocellular carcinoma (27), lung adenocarcinoma (28), and cervical cancer (29), et al. In comparison to these findings, our highlights and ideas included using the LASSO advanced algorithm to eliminate extra genes and investigating the relationship between hypoxia and the immune microenvironment, m6A-related genes, immune checkpoints, and incorporating multiple tumor-infiltrating immune cells and validity through experiment.

Our risk model is made up of four HRGs (*ANXA2, AKAP12, NR3C1*, and *GPI*), the majority of which are strongly up-regulated (**Table 3**). *ANXA2* is detected in a variety of malignancies and regulates apoptosis, metastasis, cell proliferation, invasion, adherence, and tumor neovascularization, all of which are important in tumor progression (30). The inhibition of *ANXA2* also inhibits tumor cell growth, metastasis, and survival (31). As a critical regulator of glucocorticoid hormone effects, *NR3C1* can alter gene expression in target cells and tissues, potentially leading to cancer (32). *AKAP12* controls cell signaling pathways and accelerates oncogenic development in cancer (33). *AKAP12* is a major HIF target gene that controls endothelial functional responses. Furthermore, *AKAP12* loss and gain of function studies revealed the reliance of *AKAP12* on the management of microvascular endothelial tube growth, in which it works as a “braking” mechanism for angiogenesis (34). According to new data, *GPI* is profoundly linked with a range of malignancies, and it might be exploited as a biomarker for cancer treatment (35). According to Huang et al., *GPI* might potentially be employed as a novel biomarker for GC prognosis, and it might be helpful in the diagnosis and treatment of GC patients (36). However, less study has been done on these RNAs in endometrial cancer. These findings add to our understanding of the relationships between tumor progression and clinical outcome.

**Table 3:** The full name, summaries and pathways of 4 hypoxia-related genes (HRGs) in endometrial carcinoma.

HRGs	Full name	Summaries	Pathways
ANXA2	Annexin A2	regulate cellular growth and signal transduction pathway, correlate with resistance to treatment against various cancer forms	Innate Immune System Ca, cAMP and Lipid Signaling Tyrosine Kinases / Adaptors
AKAP12	A-Kinase Anchoring Protein 12	associate with protein kinases A and C and phosphatase, and serves as a scaffold protein in signal transduction	Activation of cAMP-Dependent PKA Activation of PKA through GPCR
NR3C1	Nuclear Receptor Subfamily 3 Group C Member 1	encode glucocorticoid receptor as a regulator of transcription factors, involve in inflammatory responses, cellular proliferation, and differentiation	Glucocorticoid Receptor Signaling MIF Mediated Glucocorticoid Regulation
GPI	Glucose-6-Phosphate Isomerase	encode a member of the glucose phosphate isomerase protein family, as a lymphokine that induces immunoglobulin secretion and a tumor-secreted cytokine and angiogenic factor	Glycogen metabolism Glycolysis and gluconeogenesis

We applied GO, KEGG, and GSEA to identify pathways that are enriched in high-risk and low-risk groups. Significant enrichment of genes in hypoxia-related pathways was discovered by GO and KEGG enrichment analysis. Using GSEA, we found that most of the enrichment pathways were related to cell cycle regulation and cell division, which implies that these genes might affect tumor division and thus have therapeutic effects. Recently, it has gotten a lot of attention that N6-methyladenosine (m6A) is the most common mRNA alteration in eukaryotic cells (37). Many facets of RNA metabolism were affected by the m6A alteration, including RNA processing, nuclear export, RNA translation, and decay (37). According to new findings, m6A methylation played a critical role in endometrial cancer through a variety of pathways and expanded the potential for early cancer detection and therapy (38–40).

In the past years, tumor immune checkpoints have been associated with immune evasion and tumor microenvironment in a rising number of investigations (41). Immune checkpoints might be utilized to treat cancer, and inhibitors that block key molecules are seen to be useful in cancer treatment. Immune checkpoint blockade therapy has been demonstrated to be useful in some cancers but has had little effect on UCEC. As a result, it has been proposed that the primary immune checkpoint molecules associated with UCEC immunosuppression are probably *PD1*, *PDL1, CTLA4*, *LAG3*, and *TIM3* (41–46). According to our findings, associated immune checkpoint molecules were not significantly enhanced in the high hypoxia risk group. Ultimately, we found that only *LAG3* is the most meaningful checkpoint for the immunosuppressive environment in UCEC under hypoxic conditions. Blocking this checkpoint might have significant therapeutic implications for endometrial cancer patients.

Hypoxia can alter the cellular components and impair immune cell function, resulting in tumor growth either directly or indirectly. Impaired immune cell activity is a key component of the tumor immunological microenvironment, such as natural killer (NK) cells. NK cells are known as the immune cell system’s toxic lymphocytes. According to research, the expression of natural killer group 2 member A (NKG2A) of the NK cell receptor is up-regulated in the peripheral blood of colon cancer patients, and the monitoring and killing capabilities of NK cells are blocked, resulting in the immune escape of colon cancer cells (47). Patients were also shown to have considerably lower numbers of NK cells than normal participants, which might be a factor in the development of UCEC. These findings imply that using these activator receptors to reactivate NK cells might be a potential target for cancer treatment (11). The following two types of CD4+ T cells can be differentiated from naive CD4+ T cells: Th1 cells secrete IL-2 and IFN-, which stimulate macrophages and CD8+ T-cell proliferation and promote cell-mediated immune responses (48).

Macrophages, a key element of the TME, promote metastasis, invasion, immunosuppression, and angiogenesis, all of which contribute to cancer (49). In the TCGA database, we found substantial variations in the amount of activated M0 macrophages between high- and low-risk groups. According to the findings of this study, hypoxia can attract immune cells into the TME. TAMs are formed when blood macrophages respond to tumor signals and are classified into two types: M1 and M2. The polarization of macrophages is important in carcinogenesis. Indeed, M1-polarized macrophages (traditional activation) inhibit cancer progression and spread, whereas M2-polarized macrophages (alternative activation) enhance it (50). Hypoxia-induced cancer factors including IL-10 and TGF-beta can cause tumor-associated macrophages to develop into M2 macrophages, which have immunosuppressive properties (51). When inflammatory substances like interferon-gamma and lipopolysaccharide excite monocytes, they activate M1 macrophages, which can emit inflammatory factors like IL-6 and tumor necrosis factor-alpha and phagocytize invading infections and tumor cells (52). Hypoxia and cell death in tumor tissue generate significant quantities of cell debris and cause the release of inflammatory factors that attract and polarize macrophages and monocytes. Macrophages release inflammatory substances after polarization (52). Cancer-associated neutrophils can cause tumor suppression as well as tumor growth (53). It is well established that hypoxic TME promotes neutrophil engagement in tumors by regulating their adhesion to epithelial cells (54). HIF1 and HIF2 have both been proven to improve the survivability and functionality of neutrophils (55). Follicular helper T (Tfh) cells are a special kind of CD4+T cell that help to create germinal centers (GCs) and boost B cell responses, both of which are necessary for the creation of high-affinity antibodies to kill invading pathogens (56). Patients with high-risk scores showed larger proportions of neutrophils and mast resting cell morphologies, according to CIBERSORT. Immunosuppressive cells, such as follicular helper T cells and CD8 T cells, were increased in the low-risk group, indicating that the two groups had different levels of immunological impairment.

Based on the expression of HRGs, we built a 4-gene-based hypoxia risk model that accurately predicts the prognoses of patients with UCEC. Our model described how hypoxia status alters the immune microenvironment in UCEC patients and acted as an independent predictive factor for UCEC patients. The connection between hypoxia and the immune cells of tumors is undeniably intricate, and more independent factors and functional tests are needed to conduct a more thorough investigation. The HRGs score performs well in determining biological state and predicting UCEC survival. However, our research still has many shortcomings and flaws. First, there are no more basic experiments to demonstrate the effect of hypoxia on the prognosis of endometrial cancer. Second, the mechanism by which hypoxia affects cancer progression has not been revealed. Therefore, more research by well-designed experiments is needed.

## Conclusion

In our study, we have built a hypoxia-related gene risk score model consisting of *ANXA2, AKAP12, NR3C1*, and *GPI*. The risk score was positively and significantly correlated with the infiltration abundance of immune cell types, suggesting a close and strong relationship between the hypoxia microenvironment and tumor immune activity. This predictive model provides a novel prognostic signature for endometrial carcinoma patients and may improve treatment strategies for individuals.

## Data availability statement

The original contributions presented in the study are included in the article/**Supplementary Material**. Further inquiries can be directed to the corresponding author.

## Author contributions

JC, GW, DHZ, and XL completed data download, data collation, data analysis and results discussion. JC, DZ and YZ wrote and revised the paper. All authors contributed to the article and approved the submitted version.
