# Supplementary figures and images for: Construction of novel hypoxia-related gene model for prognosis and tumor microenvironment in endometrial carcinoma
Source: Front Endocrinol (Lausanne). 2022 Dec 15;13:1075431. doi: 10.3389/fendo.2022.1075431 (PMC9797861; doi:10.3389/fendo.2022.1075431)

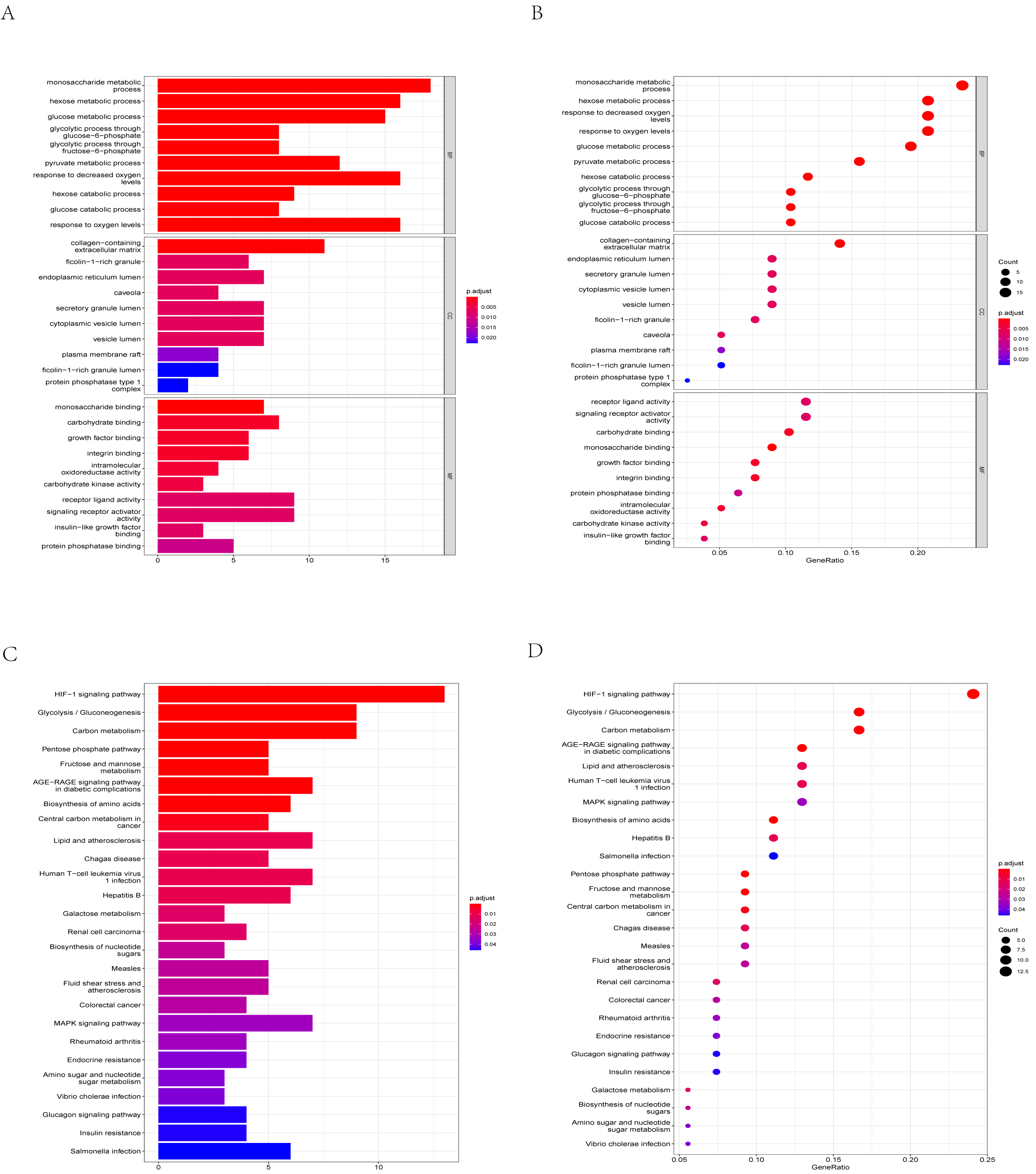

Supplement: Supplementary file 1 [file Image_1.tif]

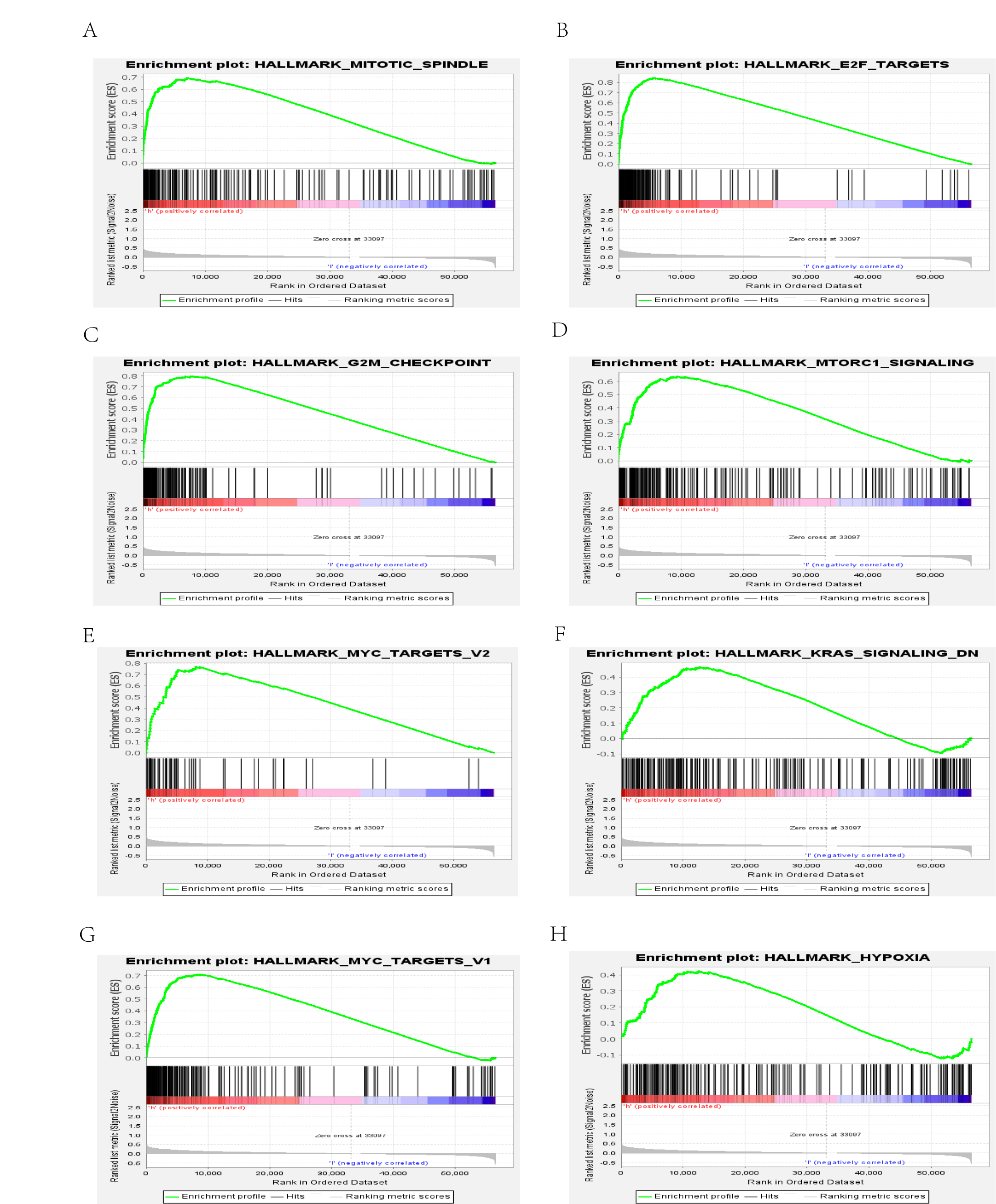

Supplement: Supplementary file 2 [file Image_2.tif]

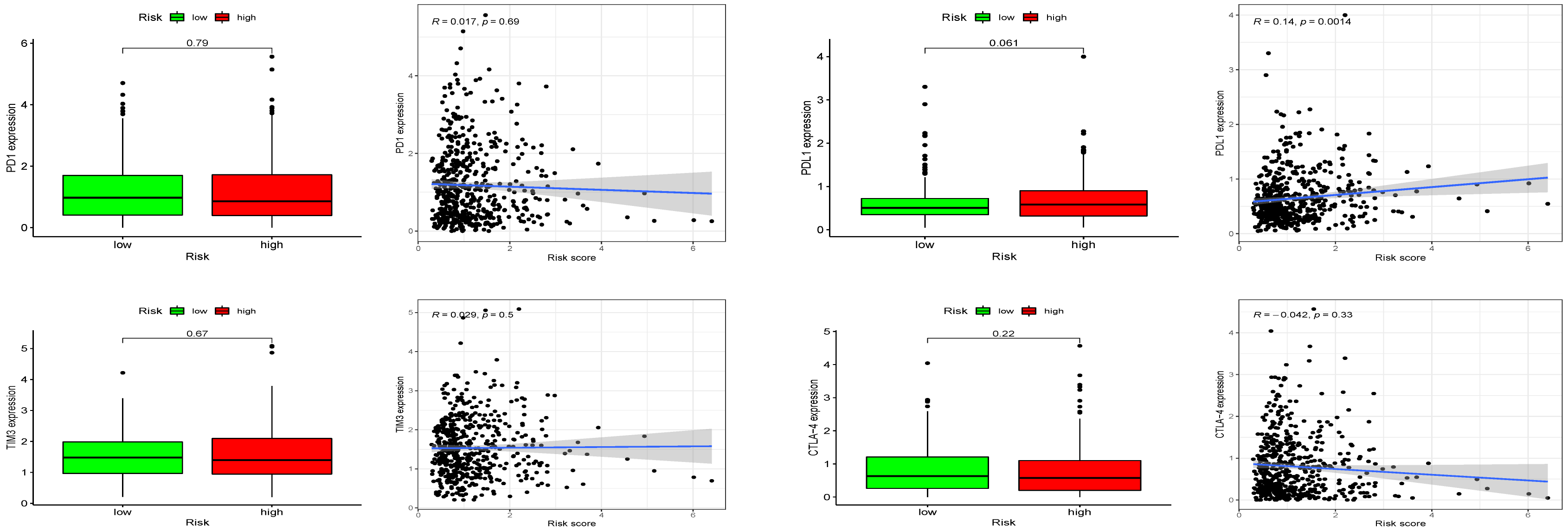

Supplement: Supplementary file 3 [file Image_3.tif]
