# Supplementary material for: Construction of novel hypoxia-related gene model for prognosis and tumor microenvironment in endometrial carcinoma
Source: Front Endocrinol (Lausanne). 2022 Dec 15;13:1075431. doi: 10.3389/fendo.2022.1075431 (PMC9797861; doi:10.3389/fendo.2022.1075431)
Supplement: Supplementary file 4 [file Table_1.docx]

| id | coef | HR | HR.95L | HR.95H | P-value |
| --- | --- | --- | --- | --- | --- |
| ANXA2 | 0.33952 | 0.71211 | 0.54701 | 0.92705 | 0.01164 |
| NR3C1 | 0.42301 | 1.52655 | 1.11222 | 2.09524 | 0.00884 |
| AKAP12 | 0.17080 | 1.18626 | 1.0011 | 1.40569 | 0.04856 |
| GPI | 0.40325 | 1.49668 | 1.10093 | 2.03467 | 0.01006 |

Supplementary Table 1 | Multivariate COX regression analysis results of the 4 hypoxia-related genes.
