# Supplementary material for: Construction of novel hypoxia-related gene model for prognosis and tumor microenvironment in endometrial carcinoma
Source: Front Endocrinol (Lausanne). 2022 Dec 15;13:1075431. doi: 10.3389/fendo.2022.1075431 (PMC9797861; doi:10.3389/fendo.2022.1075431)
Supplement: Supplementary file 5 [file Table_2.docx]

Supplementary Table 2 | Independent prognostic analysis by Univariate Cox and Multivariate Cox regression analyses.

| Clinical characteristics | Univariate analysis | | Multivariate analysis | |
| --- | --- | --- | --- | --- |
|  | p-value | Hazard ratio (95% CI) | p-value | Hazard ratio (95% CI) |
| age>65 | 0.016 | 1.683 (1.101-2.575) | 0.102 | 1.429 (0.932-2.190) |
| grade | <0.001 | 2.582 (1.795-3.714) | <0.001 | 2.121 (1.455-3.092) |
| stage | 0.397 | 1.092 (0.891-1.340) | - | - |
| riskScore | <0.001 | 1.666 (1.391-1.995) | 0.004 | 1.384 (1.110-1.724) |
